# Rethinking deep geological disposal: Opportunities for reimagining nuclear waste management created through iMAGINE

**DOI:** 10.1371/journal.pone.0359972

**Published:** 2026-10-26

**Authors:** Lakshay Jain, Omid Noorikalkhoran, Elfriede Derrer-Merk, Bruno Merk

**Affiliations:** 1 University of Liverpool, School of Engineering, Liverpool, United Kingdom; 2 University of Liverpool, School of Physical Sciences, Liverpool, United Kingdom; University of Alberta, CANADA

## Abstract

Long-term disposal and management of nuclear waste is one of the major hurdles for nuclear energy. Deep geological disposal, an idea originating in the 1970s, is currently the preferred path being followed in many countries. However, acceptance of this approach is hindered by societal concerns around the safety of the “one-million years” geological disposal and passing the waste burden to future generations. iMAGINE, an integrated nuclear system based on molten salt fast reactor technology with self-sustained iso-breeding, online clean-up and reverse reprocessing, has been proposed as an innovative way to eliminate the demand for a long-term high-level waste disposal. In this work, we investigate the use of iMAGINE as a technological approach for reimagining high-level nuclear waste management by overcoming the challenges of classical partitioning and transmutation (P&T). After discussing the current status and limitations of P&T along with an overview of the nuclear waste classification and management framework existing in major nuclear power producing countries, we demonstrate the potential impact of iMAGINE in simplifying the disposal of high-level waste. The results indicate that among the key long-lived radionuclides, many lie well below the criteria for requiring deep geological disposal. Besides Finland, India and Russia where most (or all) of the important long-lived fission products exceed current high-level waste thresholds, only two to three fission products (generally with half-lives from 30 to 100 years) exceed the national limits in Canada, China, France, Germany, Japan, and South Korea, while concrete conclusions can’t be drawn for the UK and US based on available information. The results presented here do not aim to make inflated claims but should be seen as the scientific basis for deeper discussions amongst all nuclear waste disposal stakeholders to explore a novel technological solution for rethinking high-level waste management and possibly eliminating the need for a “one-million years” geological disposal.

## Introduction

The growing need for phasing out fossil fuel-based sources to reduce greenhouse gas emissions while ensuring resilience of electricity grids in the net-zero world has led to a renewed global interest in nuclear power [1–5] and advanced nuclear technologies [6,7]. The core reason for this decision is that nuclear energy is currently the only 24/7, low-carbon system available on-demand to complement the intermittence of renewable technologies [8]. Nuclear can deliver flexibly on the energy trilemma – ensuring access to affordable, reliable, sustainable, and modern energy for all [9]. In addition, it can act as the baseload source to ensure sufficient grid inertia and stability, a fact which has been recognized since a long time [10] but came recently into sharp focus due to the April 2025 blackout in Spain and Portugal [11,12].

Despite this growing recognition and need for expanding deployment of nuclear power (see the COP28 declaration to triple nuclear capacity by 2050 [2]) along with its proven techno-economic benefits, there are some key concerns which have hampered public acceptance of this technology [13,14]. Whilst the benefits include low harmful emissions, high reliability, low operational costs, and high energy density, the disadvantages are environmental damage, waste disposal, limited resources, and long and expensive construction along with societal concerns around nuclear waste, accidents and proliferation [15]. Moreover, in countries like Germany and Austria, ideological factors rooted in cultural values [16] – often resembling religious attitudes – have contributed to strong anti-nuclear movements and political decisions to abandon nuclear power [17,18]. Among these, we have identified public anxiety about nuclear waste disposal and passing its burden to future generations as one of the major societal development barriers [15].

Currently, geological disposal is the only viable option foreseen for the management of high-level nuclear waste and originates back to 1970s. It is expected that geological disposal facilities would be able to isolate high-level nuclear waste from the biosphere for “one-million years” and more. As per current estimates, these facilities are expected to cost hundreds of billions of dollars and have faced decades-long delays in most countries due to the lack of public acceptance. This problem, and the way forward, was already distinctly highlighted in IAEA Bulletin No. 39, 1997 [19] by Viktor Arkhipov (Consultant at the Division of Nuclear Power and the Fuel Cycle, IAEA at the time) in the following words:

*“One of the greatest challenges in the use of nuclear energy is the* ***highly radioactive waste*** *… progress in the disposal of radioactive waste has been* ***influenced, and in many cases delayed, by public perceptions about the safety of the technology****. …* ***Problems of perception could be reduced*** *to an essential degree if there were a* ***way to burn or destroy the most toxic long-lived radioactive wastes*** *during the production of energy.”* [19]

In fact, effective solutions for nuclear waste management were anticipated by scientists from the outset of development of nuclear technologies, as highlighted by this excerpt from *Now It Can Be Told: The Story of The Manhattan Project* [20]:

“We always thought that it would be possible by **intensive research to eliminate much of this radioactive problem** in the future. We also hoped to **recover the Uranium remaining in the existing solutions and to reduce the bulk of the radioactive waste materials**, thus making them easier to handle.” [20]

Closed fuel cycle with partitioning and transmutation (P&T) has been researched to address these issues, with the core aims being:

- To recover and increase Uranium utilization; and
- To separate and burn Plutonium and minor actinides

The overarching idea was to avoid the demand for the “one-million years” geological final disposal.

Solving both these issues has long been seen as the holy grail for minimizing the nuclear waste challenge. The proponents of P&T promised the technology as an alternative technical solution to the long-term final disposal for high-level waste [21,22]. This led to serious efforts for development and demonstration of P&T technology beginning in the 1990s with the OMEGA program in Japan [23,24] and the CAPRA/CADRA project in France [25,26]. These were followed over the next few decades by several pan-European projects like EUROTRANS [27], EUROPART [28], GENIORS [29], PATRICIA [30], etc. These projects successfully demonstrated different steps of P&T at lab-scale [31–36]. An overview of the modern developments regarding closed fuel cycle and waste management technologies in Russia is given in PETRUS [37,38]. However, the limited effect of P&T on the final disposal has been discussed for considerable time [39,40] and will be described in detail later.

The iMAGINE program has been developed to tackle the long-term waste management challenge by eliminating the demand for the “one-million years” high-level waste disposal, serving the societal demands and needs [41], and to avoid passing the burden to future generations. It follows the recognition that P&T technology, as proposed in the 1990s, has not yet delivered on its promise. Molten salt fast reactors combined with online clean-up present a disruptive new approach to deliver on this promise while overcoming many of the challenges of classical P&T through an integrated approach. The development of molten salt reactors had already started in the 1940s leading to the successful operation of two test reactors (the Aircraft Reactor Experiment [42] and the Molten Salt Reactor Experiment [43]), four hot-critical assemblies, and about a dozen in-pile test loops in the US [44]. The main aim was to develop a thermal breeder reactor based on the Thorium/Uranium-233 (Th/U^233^) cycle but development was later stopped as the US decided to pursue only sodium-cooled fast reactor as a breeder technology. Unlike iMAGINE, which aims to address sustainability and long-term nuclear waste management challenge, the primary development driver at this time was the production of fissile material.

The innovative iMAGINE concept, a nuclear system based on integrated chloride molten salt fast reactor technology, has been proposed to improve the sustainability, proliferation-resistance, safety and economics of nuclear technologies. It has been designed to operate on an integrated closed fuel cycle with self-sustained iso-breeding [45,46] and staggered salt clean-up through reverse reprocessing allowing elementwise fission product separation [47,48]. The aim is to not only design the reactor, but to develop a holistic system looking from *cradle-to-grave* for enhancing the fuel utilization and reducing the final disposal challenge. It can overcome some of the major challenges that hindered P&T in the following ways [15,49–51]:

- Integrated energy production and waste management making it economically attractive
- Closed fuel cycle operation without fissile material separation avoiding Plutonium economy and proliferation risks
- Using fuel accessibility during operation by separating fission products rather than fissile material to avoid the need for high separation rates and complex multi-recycling
- Eliminating the demand for the “one-million years” high-level waste disposal

To deliver on the last point, we explore elementwise fission product separation through online salt clean-up which creates novel technical opportunities for segregation of material streams based on half-life, radioactivity, or heat load, as well as serving the secondary demand of radioisotopes for medical or industrial applications. Elementwise separation opens new possibilities for conditioning, handling, storage, and disposal of different waste streams, thus, reimagining nuclear waste management. Long-term operation of iMAGINE has been investigated previously and reported in several publications [46,52–54]. These long-term operational studies prove that the system is able to deliver the second and third point – integrated closed fuel cycle operation without fissile material separation while removing only the key neutron poisoning fission products. The investigations have clearly shown that the iMAGINE system can achieve self-sustained iso-breeding allowing very high fuel burnup (~ 900 GWd/tHM) and operational times for various cases with different fuel and feed configurations, including uranium tailings from enrichment, spent nuclear fuel (SNF) and SNF with already vitrified fission products [52,53]. These studies show that iMAGINE allows almost complete fuel utilization and thus very low waste amounts per unit energy produced, and provide modelling and simulation based proof from a neutronics and reactor physics perspective [52,53].

The present work aims to investigate the use of iMAGINE as a technological approach for reducing the final disposal challenge for high-level and long-lived waste. The paper is organized as follows: The Nuclear Waste Classification and Management section provides an overview of nuclear waste classification and the international and national strategies for management of different waste classes. This is followed by a description of the codes and methods used in this work in the Codes, Models and Methods section, and a detailed discussion of the results in the Results and Discussion section. The impact of iMAGINE on the final disposal challenge in different countries based on existing regulatory framework is presented in the Country-specific Scenarios section. Finally, the conclusions have been presented in the Conclusions section.

## Nuclear Waste Classification and Management

Radioactive waste can be classified for different purposes, including regulatory control, safety considerations, engineering needs, communication, etc. In terms of the long-term disposal and management of nuclear waste, different classification schemes exist at international and national levels. All schemes, however, are defined by radiological hazard and safety perspectives associated with the waste and are based on a combination of one or more of the following factors:

- Half-life of the radionuclides in the waste
- Activity level of the waste
- Heat generated by the waste
- Dose risk from the waste

An overview of the IAEA classification and country-specific criteria are presented in the following two subsections.

### IAEA classification [55]

The IAEA uses a qualitative waste classification scheme based on long-term safety and disposal. Six waste categories, as shown in Fig 1, have been defined linking them directly to suitable disposal and long-term management options. These are as follows:

- Exempt Waste (EW): Waste below minimum thresholds for radiation protection and suitable for exemption or exclusion from regulatory control. Once cleared from regulatory control, EW is no longer considered radioactive waste.
- Very Short-lived Waste (VSLW): Waste consisting primarily of materials with very short half-lives. VSLW is suitable for storage for a few years to allow for decay and subsequent clearance from regulatory control.
- Very Low-level Waste (VLLW): Waste exceeding EW criteria but not requiring a high degree of containment and isolation. It is suitable for near-surface landfill type disposal with limited regulatory control.
- Low-level Waste (LLW): Waste exceeding clearance levels usually consisting of short-lived radionuclides with higher activity concentration as well as long-lived radionuclides with limited activity concentration. LLW requires robust containment and isolation for up to a few hundred years. It is suitable for disposal in engineered near-surface facilities.
- Intermediate-level Waste (ILW): Waste with a higher activity content and larger amounts of long-lived radionuclides (particularly α emitters) than LLW but requiring limited or no heat dissipation considerations for disposal. It is suitable for disposal in intermediate depth facilities situated a few tens to few hundred meters underground.
- High-level Waste (HLW): Waste consisting of radionuclides with a very high activity concentration requiring heat dissipation considerations for disposal. HLW may also contain large amounts of long-lived radionuclides which need to be considered for the design of the disposal facility. Currently, HLW is deemed suitable only for deep geological disposal facilities situated several hundreds of meters underground in stable geological formations.

**Fig 1:**
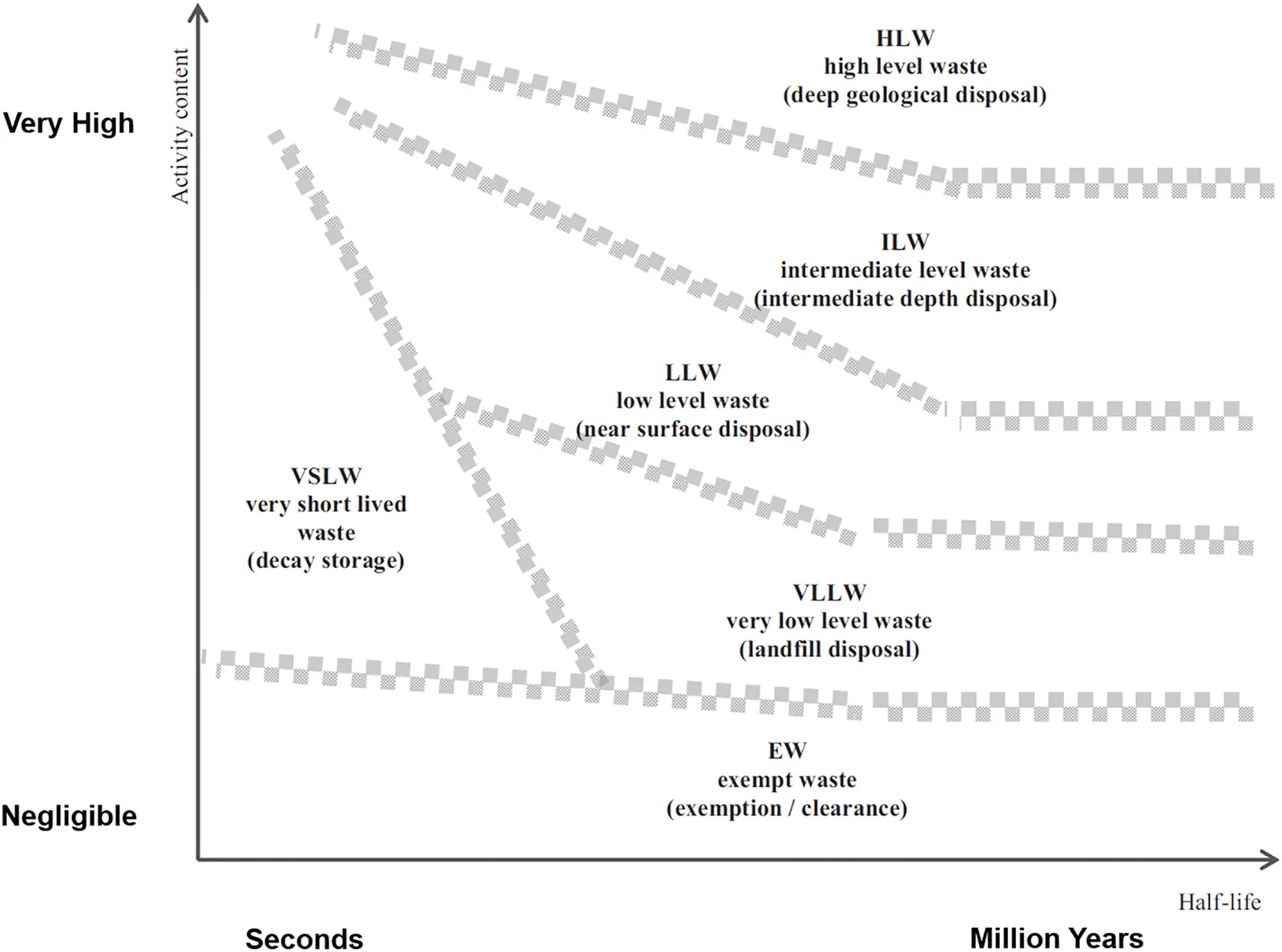
Illustration of IAEA radioactive waste classification and management scheme (adapted from [55]).

The IAEA categorization of waste is illustrated conceptually in Fig 1 where the vertical and horizontal axes represent activity content and half-life respectively. The activity can vary from negligible to very high and can be interpreted as the activity concentration (Bq/m^3^), total amount of activity (Bq), or specific activity (Bq/kg). Similarly, half-lives can range from a few seconds to millions of years and more. It is important to note that half-life of about 30 years is generally used to distinguish between short- and long-lived radionuclides since the radiological hazard associated with the former is expected to become negligible (or reduce significantly) in a few hundred years.

**Fig 2:**
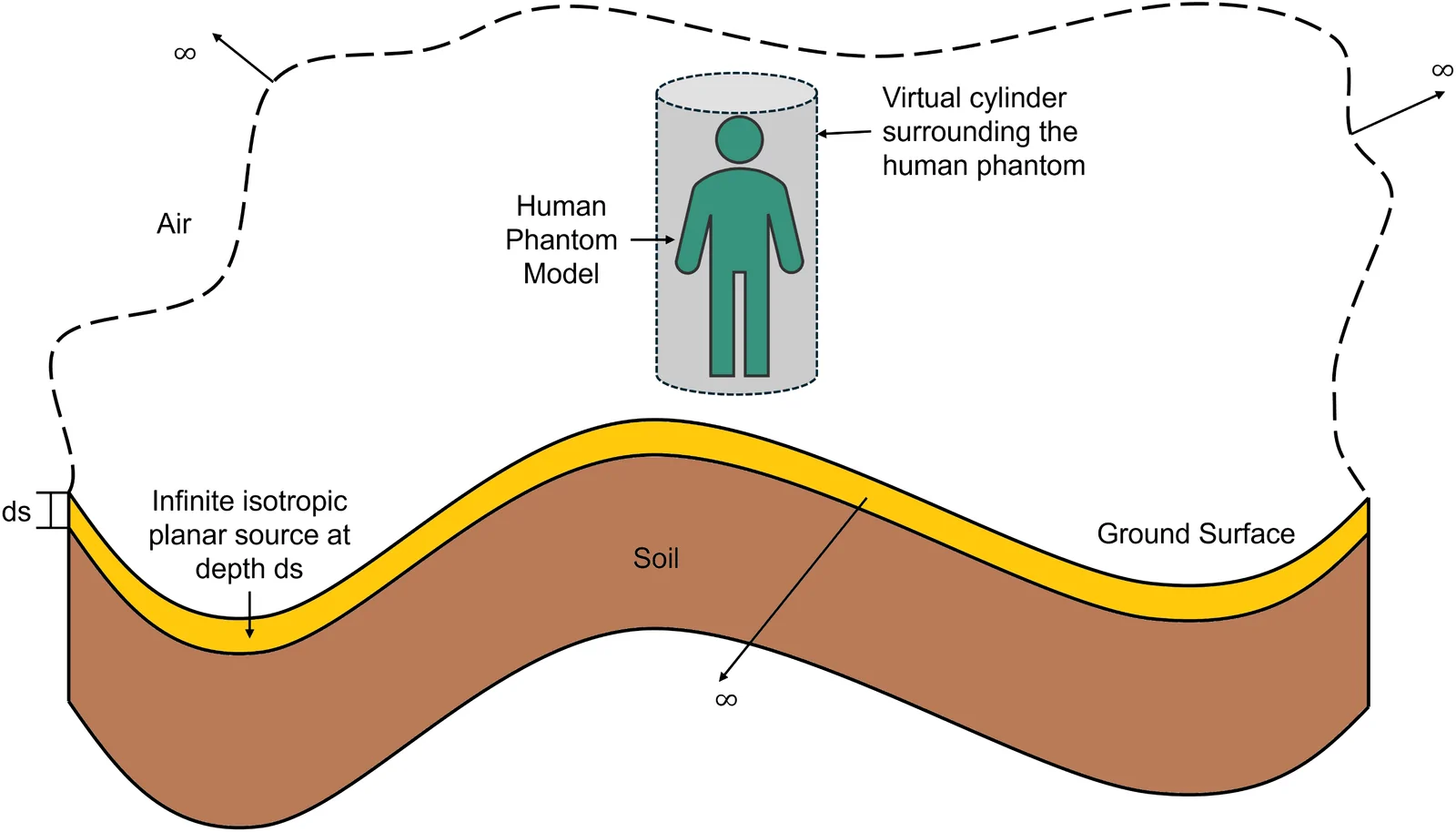
Model used in Ref. [66] to determine dose coefficients for the contaminated ground-surface exposure scenario.

### Country-specific regulatory limits and management strategies

The qualitative radioactive waste classification and management scheme developed by the IAEA is intended as an overarching guiding framework for developing detailed, quantitative national regulatory guidelines after considering regional (sometimes even site-specific) conditions. Various countries have derived their own classification system based on the same or different waste classes. Although the exact terminology may vary, classification system of each country can be broadly mapped to the waste categories described earlier in the IAEA classification subsection. The country-specific waste classification criteria (mapped to IAEA waste classes) for some of the major nuclear power generating countries has been summarized in Table 1. VSLW has not been used in Table 1 since it decays after a few years of storage and can be released from regulatory control. Similarly, EW has not been considered as it does not lie under nuclear waste regulations.

**Table 1:** Country-wise nuclear waste classification and management systems.

Country	Principle(s)	VLLW	LLW	ILW	HLW
International (IAEA) [55]	Containment and isolation requirements, heat generation and half-life	- Exceeds clearance levels but requires limited containment/ isolation - Near-surface landfill with limited control	- Exceeds clearance levels with limited long-lived radionuclides - Near-surface engineered disposal	- Higher activity waste with larger amounts of long-lived radionuclides than LLW but little/no heat dissipation - Intermediate depth disposal (~10 – ~ 100 m)	- Contains large amounts of long-lived radionuclides - Generates significant heat - Deep geological disposal
Canada [56]	Degree of containment, isolation for short- and long-term safety and hazard potential No clearly defined numerical limits	- Above clearance levels but low hazard potential - Very low concentration of long-lived nuclides - Do not require containment/isolation - Near-surface disposal	- Above clearance levels - Limited amounts of long-lived radionuclides - Require isolation for up to few hundred years - Near-surface disposal	- Higher concentration of long-lived isotopes - Require isolation and containment longer than few hundred years - No to limited heat dissipation requirement - Intermediate depth disposal	- Activity: typically 10^4^ to 10^6^ TBq/m^3^ - Long-term isolation with significant shielding - Used nuclear fuel that has been declared as waste or significant heat generating waste (typically > 2 kW/m³) - Deep geological disposal
China^a^ [57]	Activity concentrations, half-life and heat generation	- Close to or slightly above clearance levels - Very limited activity concentration for long-lived radionuclides - Require no to limited containment/isolation - Near-surface landfill disposal	- High activity from short-lived radionuclides - Limited activity concentration for long-lived radionuclides: Nb^94^ ≤ 1 MBq/kg Tc^99^ ≤ 10 MBq/kg I^129^ ≤ 1 MBq/kg Cs^137^ ≤ 1 GBq/kg - Activity: α-emitting TRUs ($t_{\frac{\text{1}}{\text{2}}}$ > 5 years) ≤ 4 MBq/kg all others ≤ 400 TBq/kg - Near-surface (0–30 m) engineered disposal	- Significant amounts of long-lived radionuclides - Activity concentration above corresponding LLW limit for each radionuclide but ≤ 400 TBq/kg - No to limited heat generation ≤ 2 kW/m³ - Intermediate depth disposal (~10 – ~ 100 m)	- Very large amounts of long-lived radionuclides - Activity > 400 TBq/kg - Significant heat generation > 2 kW/m³ - Deep geological disposal
Finland^b^ [58]	Activity concentrations, half-life and waste origin	- Activity: average ≤ 100 kBq/kg total ≤ 1 TBq total-α ≤ 10 GBq - Near-surface disposal	- Activity ≤ 1 MBq/kg - Short-lived if activity ≤ 100 MBq/kg after 500 years - Long-lived if activity > 100 MBq/kg after 500 years - LILW disposal facility at nuclear power plant site	- Activity: 1 MBq/kg to 10 GBq/kg - Short-lived if activity ≤ 100 MBq/kg after 500 years - Long-lived if activity > 100 MBq/kg after 500 years - LILW disposal facility at nuclear power plant site	- Activity ≥ 10 GBq/kg - All spent fuel - Deep geological disposal
France^a^ [59]	Activity concentrations, half-life and heat generation	- Activity ≤ 100 kBq/kg	- Activity: short-lived ($t_{\frac{\text{1}}{\text{2}}}$ < 31 years) ≤ 1 GBq/kg long-lived ($t_{\frac{\text{1}}{\text{2}}}$ ≥ 31 years) ≤ 100 MBq/kg - Surface disposal	- Activity: short-lived ($t_{\frac{\text{1}}{\text{2}}}$ < 31 years) ≤ 1 GBq/kg long-lived ($t_{\frac{\text{1}}{\text{2}}}$ ≥ 31 years) ≤ 1 TBq/kg - No to limited heat generation - Surface or near-surface disposal	- Activity ≥ 1 TBq/kg - Significant heat generation - Deep geological disposal
Germany^c^ [60]	Heat generation	- No formal VLLW definition	- Negligible heat waste with < 3 K rise in temperature at disposal chamber wall^^^ - Non-heat generating waste repository	- Negligible heat waste with < 3 K rise in temperature at disposal chamber wall^^^ but higher activity - Non-heat generating waste repository	- Heat-generating waste > 3 K rise in temperature at disposal chamber wall^^^ - Heat generating waste repository
India^d^ [61]	Solid waste: surface dose rate on waste package, activity concentration and radiation type Liquid and gaseous waste: activity concentration	Solid: - Category I waste with dose rate^e,*^ ≤ 2 mGy/h for β/γ emitters, - Activity: α ≤ 100 kBq/kg β/γ ≤ 10 MBq/kg - Near-surface disposal in earth, stone/brick lined trenches	Solid: - Category II waste with dose rate^e,*^ ≤ 20 mGy/h for β/γ emitters, - Activity: α ≤ 1 MBq/kg β/γ ≤ 100 MBq/kg - Near-surface disposal in RCC trenches/vaults	Solid: - Category III waste with dose rate^e,*^ ≤ 0.5 Gy/h for β/γ emitters, - Activity: α ≤ 4 MBq/kg β/γ ≤ 2.5 GBq/kg - Near-surface disposal in RCC trenches/vaults	Solid: - Category IV waste with dose rate^e,*^ > 0.5 Gy/h for β/γ emitters, - Activity: α > 4 MBq/kg β/γ > 2.5 GBq/kg - Deep disposal facilities
Japan^f^ [62]	Activity level and half-life	- Cat-2 waste suitable for near-surface trench disposal with no long-lived or α-emitting radionuclides - Short-lived with $t_{\frac{\text{1}}{\text{2}}}$ < 31 years - Activity: Co^60^ ≤ 10 MBq/kg Sr^90^ ≤ 10 kBq/kg Cs^137^ ≤ 100 kBq/kg	- Cat-2 waste suitable for near-surface pit disposal (< 70 m) with limited amount of long-lived or α-emitting radionuclides - Short and long-lived radionuclides - Activity: Co^60^ ≤ 1 TBq/kg Sr^90^ ≤ 10 GBq/kg Tc^99^ ≤ 1 MBq/kg Cs^137^ ≤ 100 GBq/kg α-emitting ≤ 10 MBq/kg	- Cat-2 waste suitable for intermediate depth disposal (~100 m) long-lived or α-emitting radionuclides - Long-lived with $t_{\frac{\text{1}}{\text{2}}}$ > 31 years - Activity: Cl^36^ ≤ 10 GBq/kg Tc^99^ ≤ 100 GBq/kg I^129^ ≤ 1 GBq/kg α-emitting ≤ 100 MBq/kg	- Cat-1 waste with high activity and long-lived radionuclides - Deep geological disposal - Long-lived with $t_{\frac{\text{1}}{\text{2}}}$ > 31 years - Activity: Cl^36^ > 10 GBq/kg Tc^99^ > 100 GBq/kg I^129^ > 1 GBq/kg α-emitting > 100 MBq/kg
Russia [63]	Activity level, half-life and heat generation	- Activity: α (not TRUs) ≤ 100 Bq/kg α (TRUs) ≤ 10 Bq/kg β/γ (not H^3^) ≤ 1 kBq/kg - Class 4 waste containing very low activity waste suitable - Near-surface disposal facilities at ground-level	- Activity: α (not TRUs) ≤ 1 kBq/kg α (TRUs) ≤ 100 Bq/kg β/γ (not H^3^) ≤ 10 kBq/kg - Class 4 waste containing low activity waste - Near-surface disposal facilities at ground-level	- Activity: α (not TRUs) ≤ 1 MBq/kg α (TRUs) ≤ 100 kBq/kg β/γ (not H^3^) ≤ 10 MBq/kg - Class 2 waste containing intermediate activity radionuclides with $t_{\frac{\text{1}}{\text{2}}}$ > 30 years - Class 3 waste containing low and intermediate activity radionuclides with $t_{\frac{\text{1}}{\text{2}}}$ > 30 years - Near-surface disposal up to 100 m depth	- Activity: α (not TRUs) > 1 MBq/kg α (TRUs) > 100 kBq/kg β/γ (not H^3^) > 10 MBq/kg - Class 1 waste requiring cooling prior to final disposal in deep disposal facilities - Class 2 waste containing high activity radionuclides with $t_{\frac{\text{1}}{\text{2}}}$ > 30 years requiring deep disposal without prior cooling
South Korea^g^ [63]	Annual dose rate, activity level, half-life and heat generation	- No formal definition and considered a subset of LILW - Exceeds clearance level annual dose rate: Individual > 0.01 mSv/y Total > 1 person-Sv/y - LILW disposal	- Exceeds clearance levels but below HLW - Activity for α-emitters ($t_{\frac{\text{1}}{\text{2}}}$ > 20 years) < 4 MBq/kg - No to limited heat generation < 2 kW/m^3^ - LILW disposal	- Exceeds clearance levels but below HLW - Activity for α-emitters ($t_{\frac{\text{1}}{\text{2}}}$ > 20 years) < 4 MBq/kg - No to limited heat generation < 2 kW/m^3^ - LILW disposal	- Activity for α-emitters ($t_{\frac{\text{1}}{\text{2}}}$ > 20 years) ≥ 4 MBq/kg - High heat generation ≥ 2 kW/m^3^ - Deep geological disposal
Sweden^h^ [63]	Dose rate on waste package, half-life and heat generation	- Small amounts of radionuclides with $t_{\frac{\text{1}}{\text{2}}}$ < 31 years - Dose rate^e^ < 0.5 mSv/h - Radionuclides with $t_{\frac{\text{1}}{\text{2}}}$ > 31 years can be present in restricted quantities	- Small amounts of radionuclides with $t_{\frac{\text{1}}{\text{2}}}$ < 31 years - Dose rate^e^ < 2 mSv/h - Radionuclides with $t_{\frac{\text{1}}{\text{2}}}$ > 31 years can be present in restricted quantities	- Large amounts of radionuclides with $t_{\frac{\text{1}}{\text{2}}}$ < 31 years - Dose rate^e^: < 0.5 Sv/h - Radionuclides with $t_{\frac{\text{1}}{\text{2}}}$ > 31 years can be present in restricted quantities	- Spent fuel with decay heat > 2 kW/m³ - Large amounts of radionuclides with $t_{\frac{\text{1}}{\text{2}}}$ > 31 years exceeding restricted quantities for short-lived waste - Dose rate^e^ > 0.5 Sv/h
UK [63]	Activity (level and/or concentrations) and heat-generation	- Subcategory of LLW with activity ≤ 4 MBq/m^3^ and activity per item < 40 kBq	- Activity: α ≤ 4 MBq/kg β/γ ≤ 12 MBq/kg, - No heat consideration	- Activity: α > 4 MBq/kg β/γ > 12 MBq/kg, - No heat consideration while designing storage or disposal facilities	- Waste with significant heat-generation due to radioactivity which must be considered while designing storage and disposal facilities - Waste not suitable for LLW disposal facilities due to chemical, physical or radiological property - Deep geological disposal
USA^i^ [63]	Civilian: Containment and isolation requirements (through concentrations of long- and short-lived radionuclides) and waste origin	- No formal VLLW but Class A contains very low-activity waste	- Classes A, B and C based on stability and intrusion protection with Class A < Class B < Class C	- Greater than Class C (GTCC) exceeding limits for Class C and not suitable for near-surface disposal	- High activity waste from reprocessing of SNF containing large fission products concentration and U/Th tailings
	DOE^j^: Activity concentrations and dose rate along with some overlaps with civilian classification		- Waste other than HLW, TRU^k^ and U/Th tailings		- Similar to civilian definition of HLW

a – Spent fuel is generally not considered waste and should be reprocessed, b – Spent fuel is considered as waste and should be permanently disposed, c – German waste classification scheme is solely based on heat generation, heat or non-heat generating, and not as LLW, ILW or HLW, d – Spent fuel is not considered waste, e – Dose rate on waste package from unshielded waste, f – Spent fuel is not considered waste and should be reprocessed but this policy is under review, g – Spent fuel is not currently considered waste pending a future decision on its disposition, h – Established waste acceptance criteria for different disposal routes but no legally defined waste classification system, i – Different classification systems for civilian and other US Department of Energy (DOE) waste, j – Spent fuel is not considered waste by US Department of Energy (DOE), k – US DOE owned waste contaminated with long-lived α-emitting transuranic isotopes with activity > 3.7 MBq/kg, ^ – Temperature rise of 3 K at disposal chamber wall for Schacht Konrad is equivalent to decay heat of ~200 W/m³, * – Equivalent Dose Rate [mSv/h] = W_R_ x Absorbed Dose Rate [mGy/h]; Radiation Weighting Factor (W_R_) for β/γ radiation = 1.

## Codes, Models and Methods

The present work aims to follow up previous studies on iMAGINE by analyzing the impact of the proposed system on long-term waste management. This is done through characterization of various fission product components in terms of their yield, radioactivity, and decay heat generation using the nuclear data library handling routines of OpenMC [64] and ENDF/B-VIII.0 nuclear data library [65]. To estimate the equivalent dose risk from different waste components, exposure to contaminated ground surface has been considered as the exposure scenario from an infinite isotropic planar source with an infinitesimally small thickness at the ground-air interface. The source concentration is calculated by assuming that the amount of radionuclide produced per unit energy is uniformly spread over an area of 1 m^2^. This is inarguably highly conservative since:

1) It represents an almost impossible contamination and/or exposure scenario where a radiologically significant material is released outside regulatory control, and
2) It assumes no shielding which is an extremely unlikely case in any radioactive waste management scheme.

Thus, the results would be highly conservative almost representing a limiting value and bounding case but would be suitable for creating understanding and facilitating the discussion. The dose coefficients used to convert radioactive source concentration to the total body and skin dose rate equivalent have been taken from US Environmental Protection Agency Federal Guidance Report No. 12 [66]. An illustration of the model used in Ref. [66] for calculating the dose coefficients is shown in Fig 2.

## Results and discussion

Serious efforts have been made previously in exploring P&T as a technological alternative to the “one-million year” final disposal, as mentioned earlier. However, it only reached a limited effect on the long-term final disposal. During a recent panel discussion on the current status of P&T at the 16^th^ Information Exchange Meeting on Actinide and Fission Product Partitioning and Transmutation [67], an expert panel concluded that many steps of P&T technology have been successfully demonstrated at lab-scale. However, despite this past progress, P&T technology has never reached industrial maturity due to various reasons (see Fig 3), including high costs, complexity, proliferation risks, and time demand. The demonstration of the systemic interplay of individual steps into a complete process and integrated system is also still lacking [49,67,68]. Moreover, the focus of P&T has always been on the reduction of radiotoxicity of nuclear waste by transmutation and burning of transuranics. Proposed approaches for the transmutation of long-lived fission products have also not progressed to a sufficiently promising stage. As a result, P&T has not been accepted by the final disposal specialists as a feasible solution. Final disposal safety cases are dominated by the high solubility of long-lived fission products, such as Selenium-79 (Se^79^) and Iodine-129 (I^129^), in groundwater. In contrast, eliminating transuranic radionuclides has minimal influence on the long-term disposal challenge due to their limited solubilities under the reducing conditions relevant in most geological repository settings [39,40]. As discussed earlier, the iMAGINE system has been designed to overcome these challenges by integrating energy production with waste management and reverse reprocessing.

**Fig 3:**
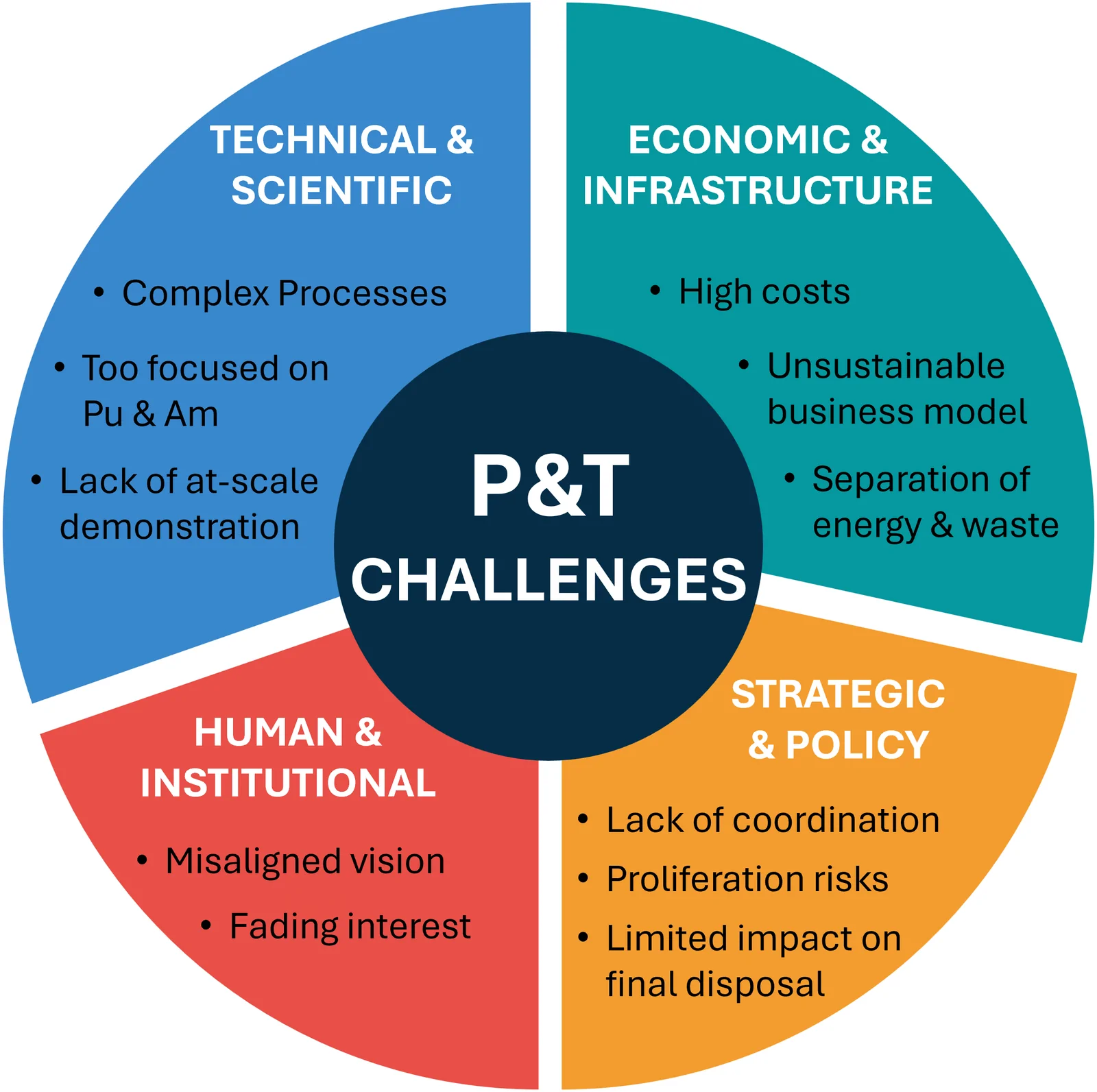
Challenges of classical Partitioning and Transmutation (P&T).

iMAGINE is based on a chloride-based molten salt fast reactor operating with a much harder neutron spectrum even compared to solium cooled fast reactors. A major fraction of the neutron flux has energy > 100 keV and the peak flux is around 500 keV [69]. Although bulk of the fission reactions would be with U^235^, Pu^239^ and Pu^241^, some contribution would also come from U^238^ and the even Pu isotopes Pu^240^ and Pu^242^, and to a small extent from the higher minor actinides. However, the fission product yields at 500 keV are quite similar for different fissioning isotopes. This is also reflected in Fig 4 which shows the cumulative heat generated from the decay of fission products from fission of 1 kg of different heavy metals, where decaying radionuclides have been binned by minimum half-life. Interestingly, the energy produced from the complete fission of 1 kg of heavy metal is ~ 22 GWh, equivalent to ~36 minutes of electricity demand for the UK in 2024 (total UK electricity demand in 2024 = 319 TWh [70]). Due to this, Pu^239^ has been chosen as the representative radionuclide for further analysis of waste properties. It must be noted that a minimum cutoff of 1 year was chosen for half-life bins since shorter lived isotopes have no contribution towards the long-term decay heat generation. It is interesting to observe from the results shown in Figure 4 that the total decay heat generated by all long-lived fission products with half-life above 30 years is only about 3.5 W/kgHM.

**Fig 4:**
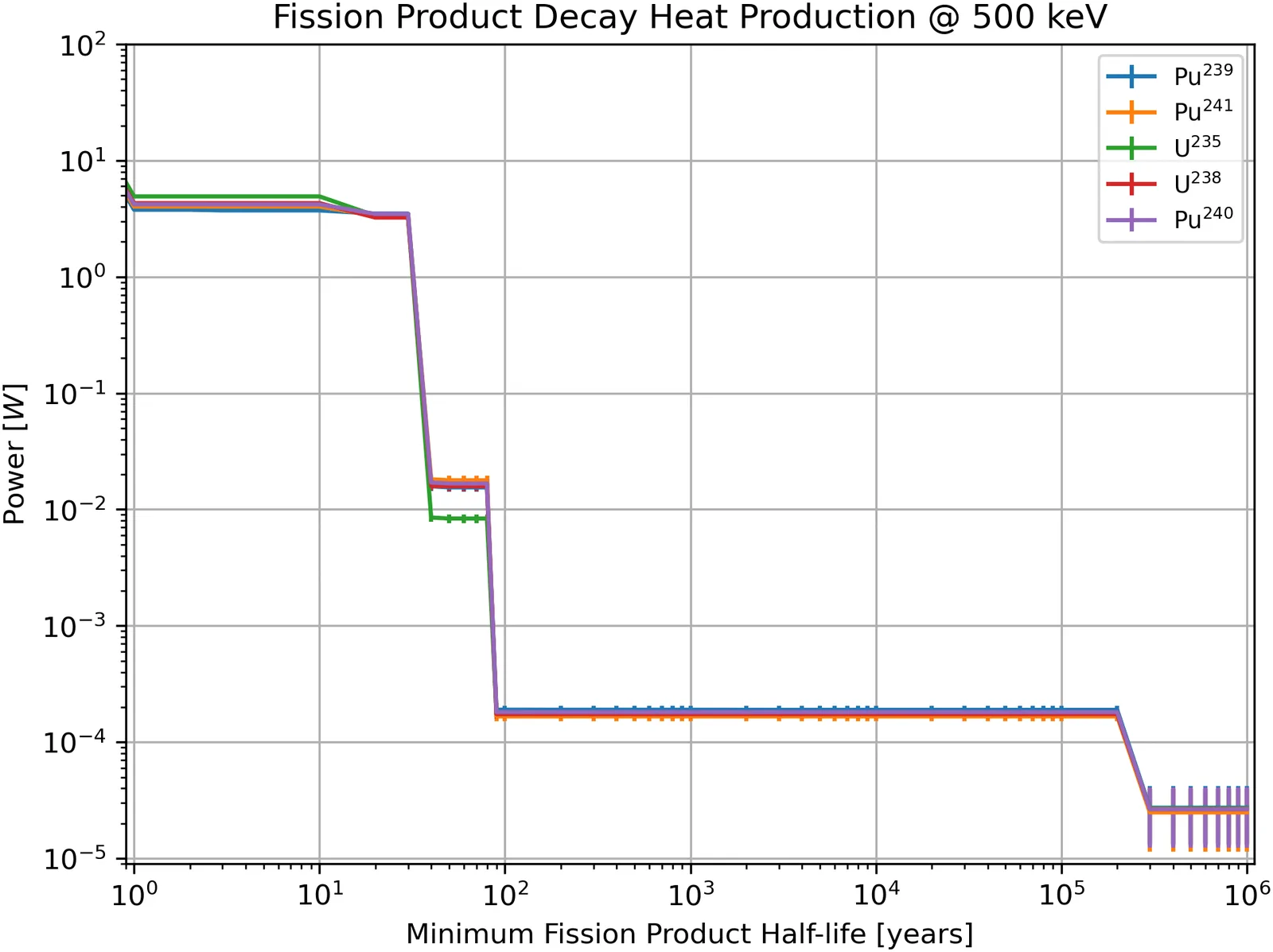
Total decay power of all fission products (binned by minimum half-life) from fission of 1 kg of different heavy metals. Error bars represent the overall uncertainty due to uncertainty in fission products yield, half-life and decay energy.

### Long-lived fission products

Fission of Pu^239^ with 500 keV neutrons leads to ~ 1250 fission products (~ 1200 to ~ 1300 for different U and Pu isotopes of interest) [65]. However, only 37 of these isotopes have half-lives > 30 years [65] and would be of interest for further analysis of long-term behaviour. Another key factor to highlight is that among these, Cs^137^ ($t_{\frac{\text{1}}{\text{2}}}$ = 30.08 years) [65] is not considered for deep geological disposal in certain countries, like France and Sweden, where minimum half-life threshold for long-lived radionuclides is 31 years. This is due to the following reasons:

- interim storage of nuclear waste for cooling and conditioning prior to being disposed of in deep geological facilities
- radioactive waste classification criteria and disposal system as defined by IAEA and waste disposal strategy followed by most countries, as mentioned earlier in the Nuclear Waste Classification and Management section
- expected 300–500 years of operational and monitoring period for most deep geological disposal facilities [71–75]

Other shorter-lived radionuclides would have decayed almost completely in this time and have no influence on the decay characteristics of the waste. Also, it must be noted here that a highly conservative approach with no interim cooling time has been considered for the radionuclides in waste. The radiological and heat hazard posed by some long-lived fission products (especially with half-lives < 100 years) would reduce if interim cooling time is used.

The decay properties of these long-lived fission products are shown in the bubble chart given in Fig 5. The half-life and the specific fission product amount are given along the horizontal and vertical axis (logarithmic scales), respectively, whereas the decay energy is represented by the bubble area and the decay type by colour. Four reference lines (100 years, time period of existence of *Homo sapiens* as a species, age of the earth and amount of isotope 0.1 µg/1 kg of fissioned Pu^239^) have also been plotted on the chart to facilitate a deeper discussion about the importance of these fission products towards deep geological repositories and their potential risk for humanity.

**Fig 5:**
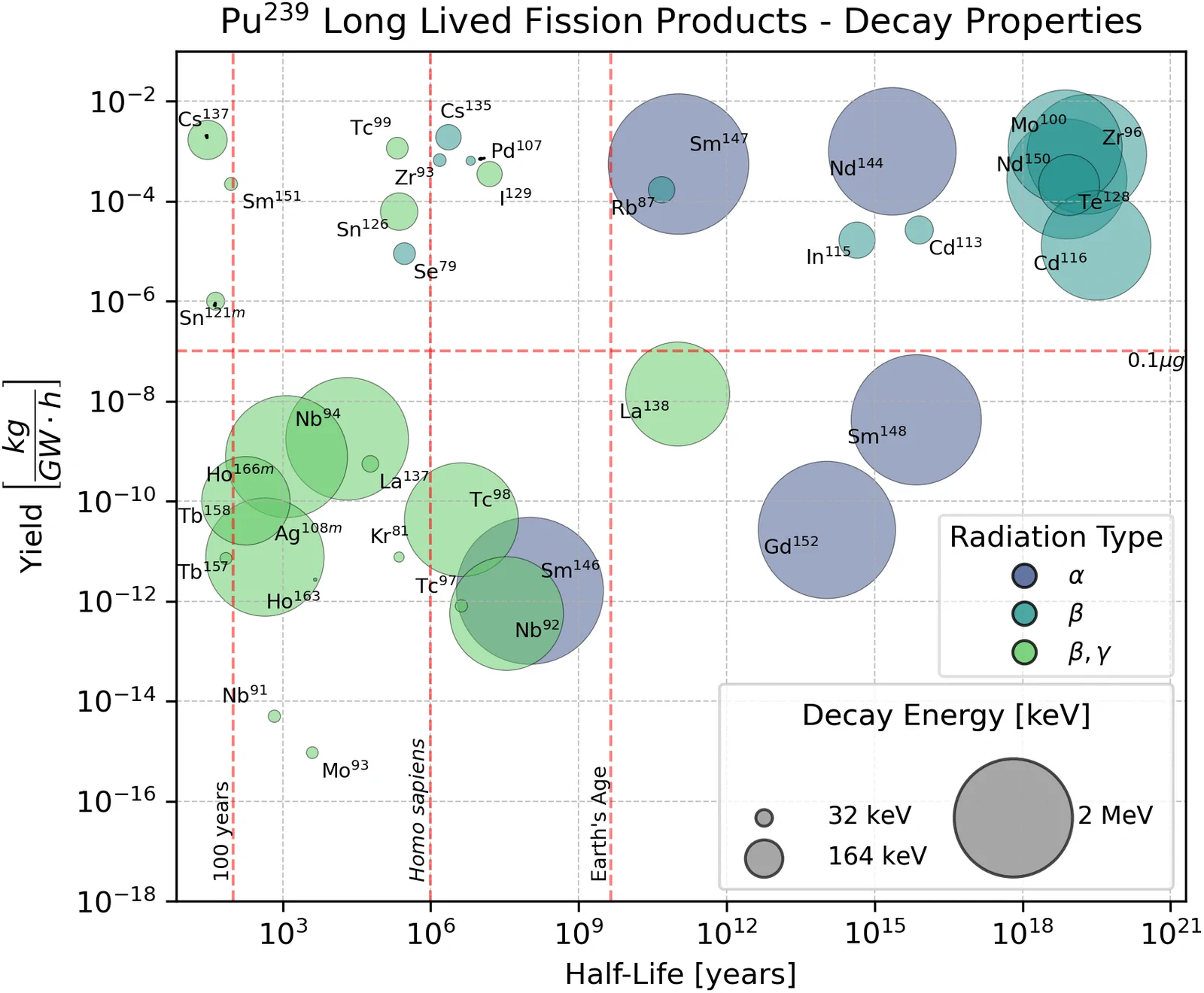
Cumulative mass yield for long-lived fission products ($t_{\frac{1}{2}}>$ 30 years) on fission of 1 kg of Pu^239^. Colour and area of the bubble represent decay type and decay energy in keV, respectively.

All radionuclides with half-lives greater than the Earth’s age (to the right of corresponding reference line) decay extremely slowly and have negligible decay heat and radioactivity contributions. Thus, these can essentially be considered as “stable” for the purpose of this analysis and can be excluded from further consideration. In fact, the half-lives are ≥ 13.8 billion years [65], the estimated age of the universe. However, the decay can be observed and measured.

Similarly, all fission products below the horizontal reference line are produced in insignificantly small amounts and may be neglected due to their negligible contributions. Moreover, the energy generation required to produce non-trivial amounts of these radionuclides would be massive. This leads to focusing on only 10 remaining radioisotopes (see Fig 6 for zoomed-in view) suitable for further analysis. These can be classified into two categories:

- 30 years $<\;t_{\frac{\text{1}}{\text{2}}}<$ 100 years – Sn^121m^, Cs^137^ and Sm^151^
- $t_{\frac{\text{1}}{\text{2}}}\gg$ 100 years – Se^79^, Zr^93^, Tc^99^, Pd^107^, Sn^126^, I^129^ and Cs^135^ [$t_{\frac{\text{1}}{\text{2}}}\;\in ($2×10^5^, 2×10^7^) years]

**Fig 6:**
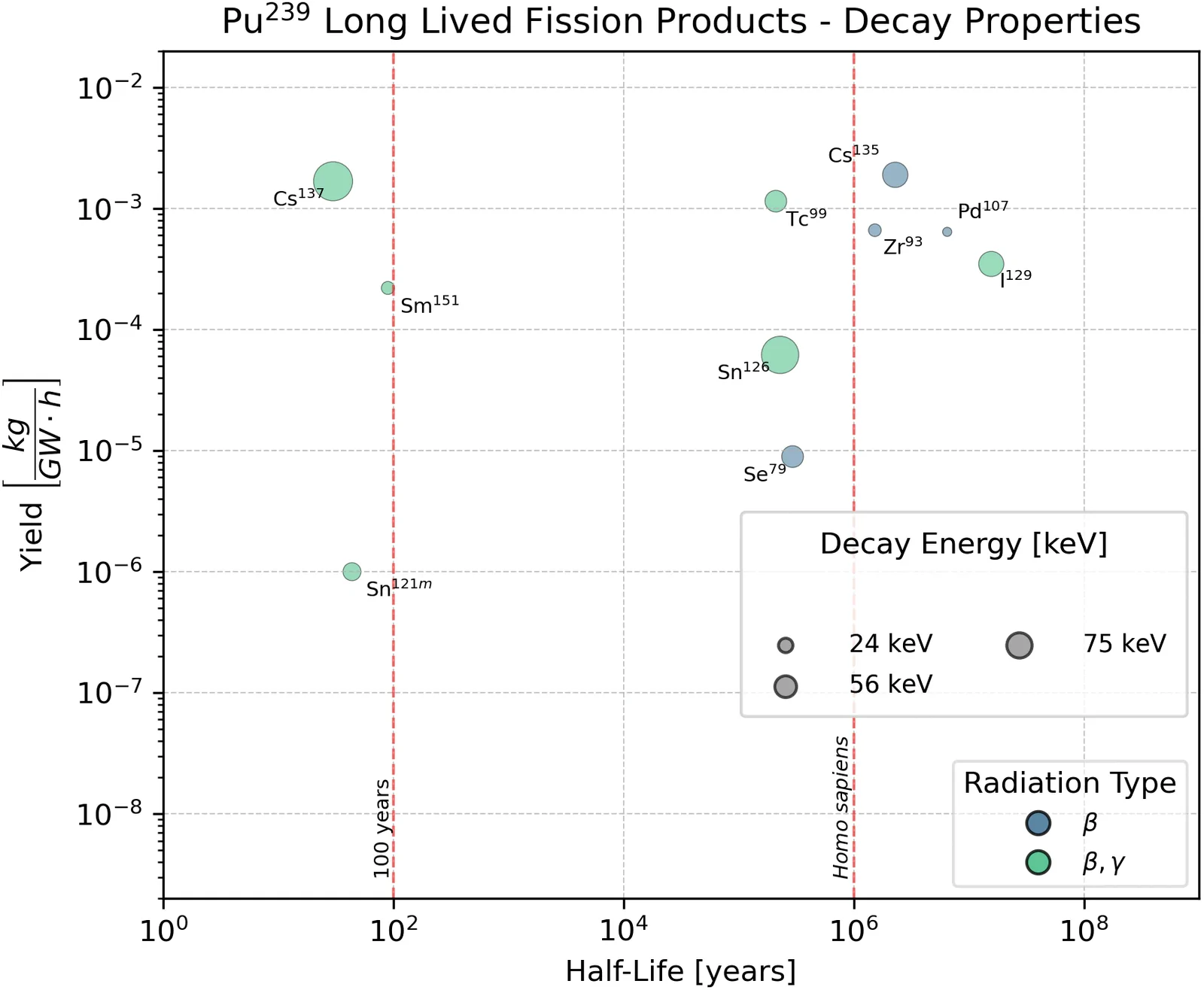
Long-lived fission products (30 years $<t_{\frac{1}{2}}<$ 10^8^ years) generated from the fission of 1 kg of Pu^239^ important for waste management considerations. Colour and area of the bubble (same scale as Fig 5) represent decay type and decay energy in keV, respectively.

It must be mentioned that all the remaining radioisotopes undergo either a β^–^ decay or a β^–^ decay followed by γ emission. Also, half-lives in the latter group, conventionally treated as the defining isotopes in the safety case of a deep geological disposal, are of the order of existence of the human species.

### Radioactivity, decay heat and dose risk from key fission products

The overview on nuclear waste classification and disposal strategies provided in the Nuclear Waste Classification and Management section indicates that there are only overarching IAEA guidelines globally and each country has its own specific classification criteria and disposal policies derived from the IAEA guidelines. The various national nuclear waste classification and management strategies are broadly based on the same qualitative scheme prescribed by IAEA and seemingly consist of a potential combination of maximum 3 primary parameters: decay heat generation, radioactivity, and dose risk. Thus, general reference thresholds have been identified for each of these parameters to facilitate further discussions (see Table 2). These reference threshold levels represent the minimum and maximum among country-specific criteria to identify which fission products necessarily require deep geological disposal, as given earlier in Table 1. It is also essential to note that most existing waste classification schemes are intended for bulk amounts of mixed radioactive waste and might not be directly applicable to purified and concentrated forms of individual radionuclides.

**Table 2:** Variation in threshold levels used by different countries for waste requiring deep geological disposal. For other countries discussed in Table 1, the threshold criterion is either inconclusive based on available information or undefined since the corresponding quantity is not used in the national scheme.

Parameter	Min. Threshold Level	Country	Max. Threshold Level	Country
Volumetric Decay Heat	200 W/m^3^	Germany	2000 W/m^3^	Canada, China, South Korea, Sweden
Specific Activity	10 MBq/kg	Russia	400 TBq/kg	China
Dose Rate	500 mSv/h	India, Sweden	500 mSv/h	India, Sweden

The volumetric decay heat (decay heat per unit volume), specific activity (activity per unit mass) and dose rate (mSv/h) were calculated using the methodology defined in the Codes, Models and Methods section and have been plotted in Fig 7. The results shown in Fig 7 clearly highlight that waste classification schemes of various countries are simply too different and thus, a uniform global evaluation leading to meaningful results is not possible. Whilst no reasonable worldwide conclusions can be drawn at once, observing the combined global limits leads to some useful insights. However, before discussing these results in greater detail, it is necessary to highlight some key assumptions:

- All quantities of interest have been calculated assuming no interim storage and cooling period. This is especially important for the fission products with half-lives ranging from 30 to 100 years which are likely to have the highest radioactivity and decay heat load. These radionuclides would decay substantially with interim storage even when only stored for a few decades, as shown in Fig 8. The figure shows the effect of interim storage (up to 50 years) on the radioactivity and decay heat load of Sn^121m^, Cs^137^ and Sm^151^. As expected, the quantities halve corresponding to the half-life of each fission product, such as ~30 years and ~44 years for Cs^137^ and Sn^121m^ respectively. Thus, the consideration of no interim storage and cooling time is highly conservative and will influence all other quantities.
- The volumetric decay heat of each fission product was calculated by using the mass yield of the corresponding radionuclide per unit energy generated (see Fig 7 top-right subplot) and its known mass density [76]. This implies that each fission product is stored without any preprocessing (for, e.g., vitrification or another way of conditioning) in a form corresponding to its theoretical density. This is a highly conservative approximation.
- The specific activity is calculated by scaling the fission product activity from the amount produced per unit energy (see Fig 7; right subplot in second row) to 1 kg of the fission product. This should be correlated with the amount of energy produced for accumulating 1 kg of the corresponding radionuclide (see Fig 9). These energy amounts can be of the order of electricity demand for an entire nation (UK electricity demand in 2024 has been used as a reference in Fig 9).
- The dose rate is calculated for hypothetical exposure to contaminated ground surface assuming the fission product produced from generation of unit energy is spread over an area of 1 m^2^ with an infinitesimally small thickness at the ground-air interface, as mentioned in the Codes, Models and Methods section. This is highly conservative since the radionuclide will be disposed of in a well-designed engineered waste container which eliminates the possibility of the assumed ground surface contamination as well as direct human exposure for disposed waste. Moreover, the dose rate thresholds correspond to those at the surface of the unshielded waste packages.

**Fig 7:**
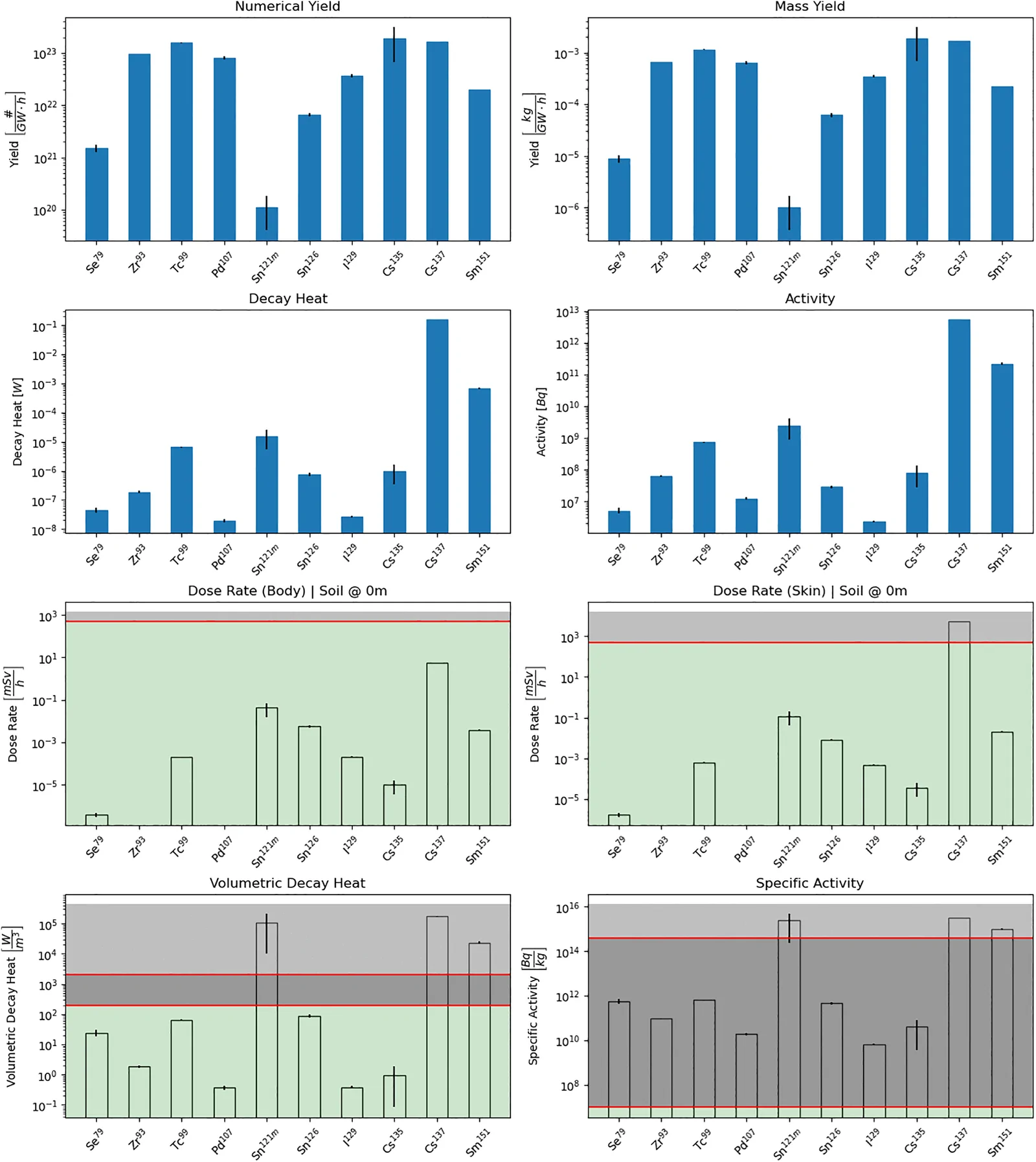
Amount, decay heat, radioactivity, and dose risk of key fission products. Error bars represent the overall uncertainty due to the uncertainty in fission products yield, half-life and decay energy. The red reference lines correspond to the worldwide minimum and maximum defined threshold criteria given in Table 2. Green represents the radionuclides below the worldwide minimum threshold criteria. Dark grey represents the radionuclides above the worldwide minimum thresholds but below the worldwide maximum thresholds and shows the variability of country-specific waste classification. Light grey represents the non-use of quantity concerned in other countries or the lack of conclusive information.

**Fig 8:**
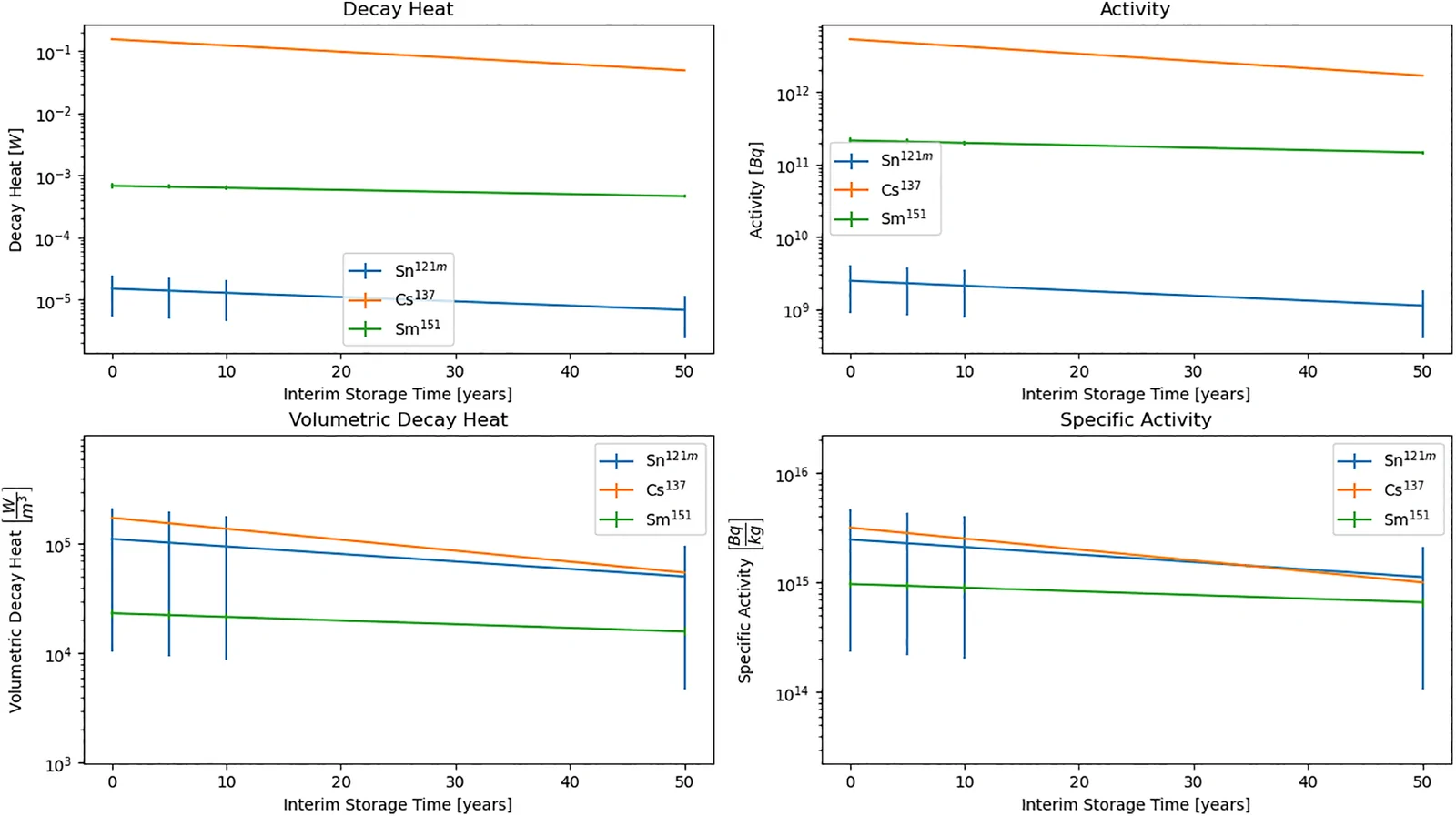
Effect of interim storage on the decay heat and radioactivity of Sn^121m^, Cs^137^ and Sm^151^. Error bars represent the overall uncertainty due to the uncertainty in fission products yield, half-life and decay energy. The large uncertainty in decay heat and radioactivity for Sn^121m^ (blue line) is primarily due to its extremely low but highly uncertain fission yield.

**Fig 9:**
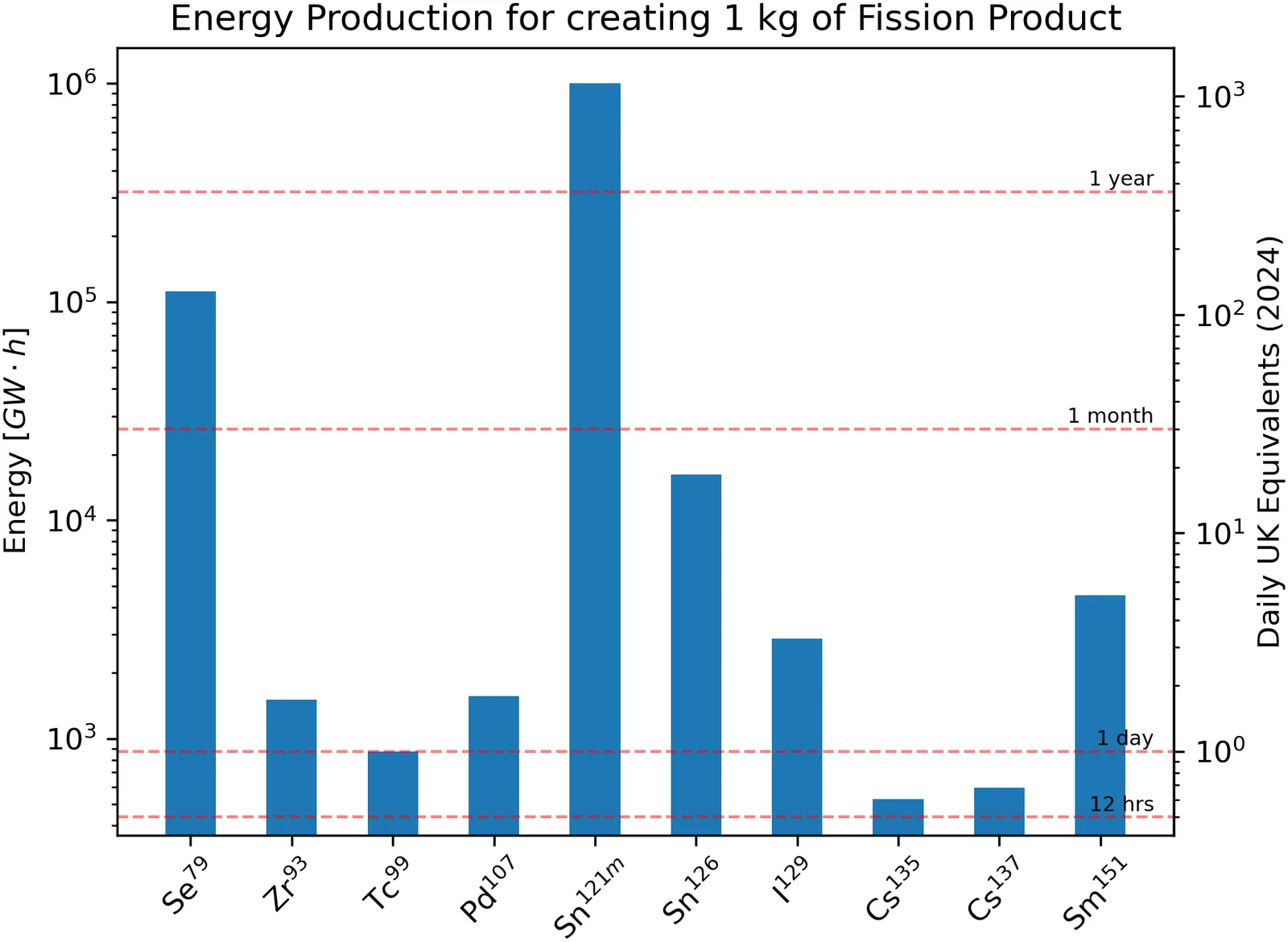
Energy generation required per unit mass of long-lived fission product accumulated. The daily equivalent of UK electricity demand in 2024 is represented on the right axis. The reference lines indicate the UK electricity requirements (based on annual demand in 2024) that would have been met for producing 1 kg of different radionuclides.

Based on the minimum threshold levels identified in Table 2 (lower reference lines in Fig 7), it can be seen that the long-lived fission products exceeding the most stringent worldwide criteria are as follows:

- Dose Rate (Body) – None
- Dose Rate (Skin) – Cs^137^
- Volumetric Decay Heat – Sn^121m^, Cs^137^, Sm^151^
- Specific Activity – Se^79^, Zr^93^, Tc^99^, Pd^107^, Sn^121m^, Sn^126^, I^129^, Cs^135^, Cs^137^, Sm^151^. However, among these, only Sn^121m^, Cs^137^, and Sm^151^ exceed the maximum specific activity limit (400 TBq/kg) among countries considered (see the upper reference line in bottom-right subplot Fig 7).

These results clearly show that a much **more detailed analysis of the country-specific regulations must be performed** to draw meaningful conclusions while the conservative assumptions used here may need to be reconsidered at a later stage. However, the results obtained here can be used to feed this more detailed analysis to identify potential national strategies which can involve specific conditioning, the definition of interim storage times before disposal, or a more detailed discussion of the validity of the limit values in the case they are significantly different to the majority of other countries. Ideally, this could lead to a harmonization of the waste criteria on the longer term.

Delving deeper into the specific activity of all the key long-lived fission products considered here, the levels seem to be extremely high and might be misinterpreted as a deal breaker at the first glance. It is important to consider the significant variation between the national limits used in different countries ranging from 10 MBq/kg, one of the most stringent used globally, and 400 TBq/kg, the highest among major nuclear countries considered in this work. Moreover, as noted earlier, energy on the scale of national electricity requirements must be generated to accumulate non-trivial quantities of even the highest yielding fission products (see Fig 9). For instance, energy equivalent of ~14–15 hours of UK electricity demand in 2024 will need to be generated for producing 1 kg of Cs^135^ and Cs^137^. This increases to the energy equivalent of ~1 day for Tc^99^, another fission product with relatively high yield. Interestingly, to produce 1 kg of Sn^121m^ and Sm^151^, which seem to be problematic radioisotopes for HLW disposal, the energy equivalent of ~3 years and a week worth of the UK electricity demand (2024) would need to be generated. Translating these values back to the operation of one or several power plants will lead to significant operational times. This must also be correlated with the highly conservative assumptions of no waste treatment, conditioning, or interim storage for cooling used here.

On the basis of the results shown, it can be concluded that only the fission products with 30 years < $t_{\frac{\text{1}}{\text{2}}}<$ 100 years are above the decay heat (Sn^121m^, Cs^137^, Sm^151^) and dose rate (Cs^137^ only for skin and not total body) thresholds of 200 W/m^3^ and 500 mSv/h, respectively. The volumetric decay heat will anyway be reduced by placing the nuclide in a suitable waste form with lower density (in most cases, the potential maximum waste loading of conditioning matrices is 30% or less) or simply through a longer interim storage time, as shown in Fig 8, before final disposal. Similarly, the surface dose rate for a well-designed engineered waste package can be controlled by the amount of shielding while exposure risk to public at ground level can be reduced by a soil layer just a few meters thick. As far as the activity is concerned, each of the 10 fission products exceed the strictest specific activity threshold level used globally (10 MBq/kg). However, as mentioned above, specific activity should be correlated with the amount of energy that must be generated to produce 1 kg of the corresponding isotope, as shown in Fig 9. Moreover, there is significant variation in the criteria used worldwide and the country-specific activity limit might vary significantly, as can be seen with only Sn^121m^, Cs^137^, and Sm^151^ exceeding the limit when considering the highest defined threshold.

## Country-specific Scenarios

Concluding from the outcomes of the previous section, the refined analysis in this section is based on the country specific data correlated with the results of the key long lived fission products provided earlier. The potential impact of iMAGINE, with its given opportunities through reverse reprocessing leading to elementwise fission product separation, on reducing the high-level waste disposal challenge will be discussed here.

The high-level waste disposal challenge of different countries can be inferred by assessing the results obtained in the previous section with respect to the waste management strategies described in the Nuclear Waste Classification and Management section. The results are presented using the infographic given in Fig 10, where blue denotes that the fission product exceeds HLW criteria and requires deep geological disposal as per the current national guidelines, green denotes no need for deep geological disposal, and grey corresponds to either inconclusive or likely based on available information. These results are based on highly conservative and simplifying assumptions used earlier. Some of the key underlying approximations are as follows:

- no interim storage and cooling time for the waste,
- no pre-conditioning or treatment of the fission product(s),
- no containment or shielding for the radionuclide(s),
- presence of the radionuclide in the concentrated bulk amounts required for the analysis, particularly when considering mass- or volume-averaged criteria such as specific activity, volumetric decay heat, etc.

**Fig 10:**
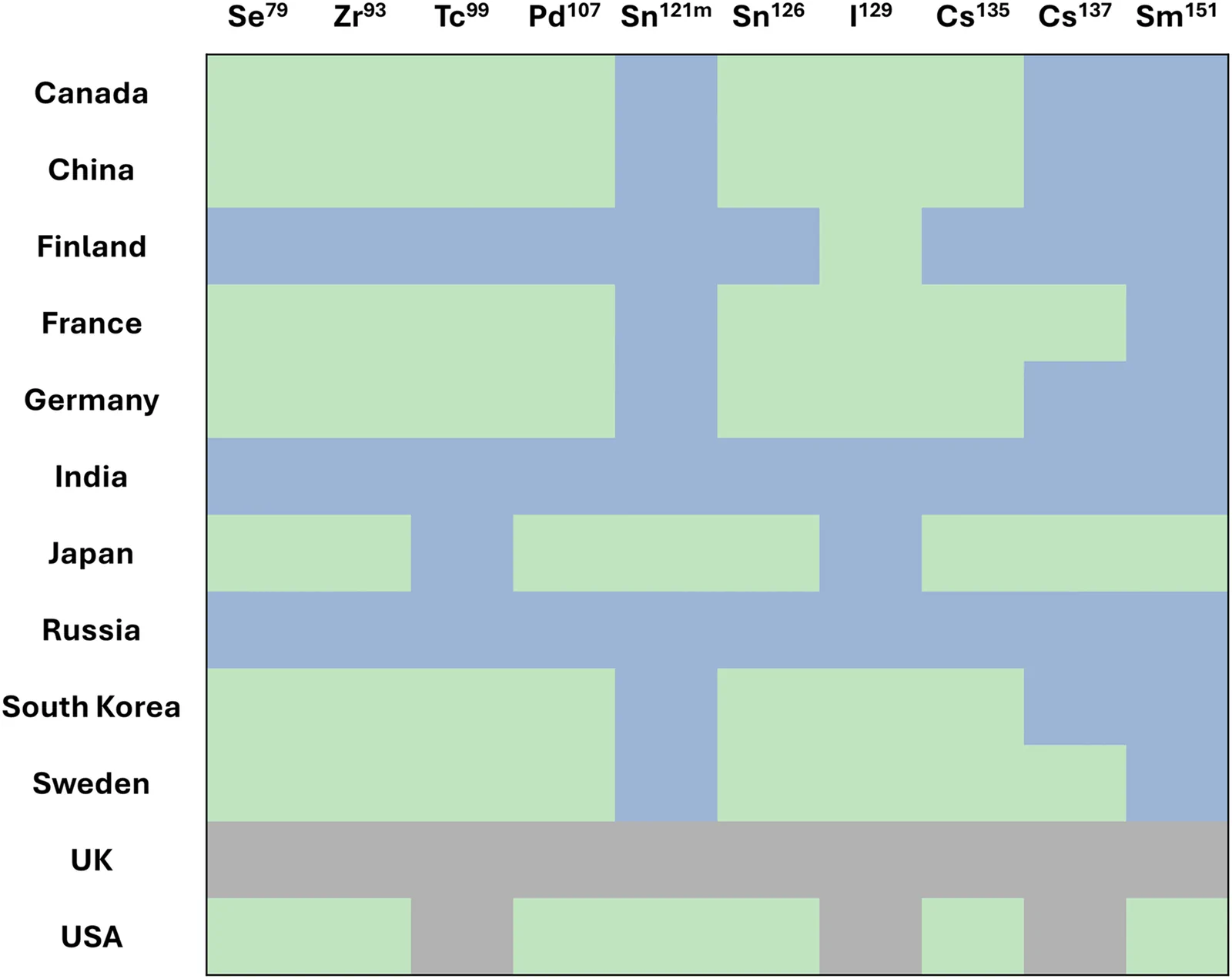
Deep geological disposal requirements for long-lived fission products in various countries based on current waste classification and management strategies at national level. Blue denotes that the fission product exceeds HLW criteria and requires deep geological disposal as per the current national guidelines, green denotes no need for deep geological disposal, and grey corresponds to either inconclusive or likely based on available information.

It must be reiterated that most existing waste classification criteria seem not to be developed or meant for purified and concentrated amounts of fission products.

Considering all the caveats, limitations, and conservative approximations, some immediate analysis gives the following picture without considering any optimization strategy. The overview given in Fig 10 indicates that all long-lived fission products identified previously would require deep geological disposal in India (specific activity and dose rate) and Russia (specific activity) based on their respective national waste management regulations and strategies. Similarly, Finnish regulations indicate that all long-lived fission products besides I^129^ exceed the HLW specific activity threshold.

However, for a large group of other countries, only Sn^121m^, Cs^137^ and Sm^151^ exceed the corresponding national HLW thresholds even under conservative assumptions. This is the case for Canada (decay heat while the numerical limits for other quantities are not clearly defined), China (specific activity and decay heat), Germany (decay heat), and South Korea (specific activity and decay heat). In the case of France (specific heat and decay heat) and Sweden (decay heat and dose rate), only Sn^121m^ and Sm^151^ need deep geological disposal since Cs^137^ with half-life below 31 years is considered short-lived as per their existing waste classification framework. Under the current Japanese regulations, only Tc^99^ and I^129^ exceed the minimum specific activity thresholds for Cat-1 waste (long-lived radionuclides with high activity, equivalent to HLW) and thus, would require deep geological disposal.

Among the long-lived fission products, Tc^99^, I^129^ and Cs^137^ would most likely be classified as GTCC waste in USA as per the radionuclide-specific volumetric activity thresholds defined by the US Nuclear Regulatory Commission [77]. Finally, it is not possible to define conclusively using available information which of the long-lived fission products would require geological disposition in the UK. Assuming the heat generation criteria used in other countries (such as 200 W/m^3^ in Germany), Sn^121m^, Cs^137^ and Sm^151^ would be classified as significant heat generating waste. Other long-lived fission products may need deep disposal if additional criteria must be satisfied.

Thus, the massive impact of iMAGINE in reducing the final disposal challenge for various countries can be clearly seen, but a final recommendation regarding the opportunity to completely avoid the “one-million years” disposal for high level waste could only be given in close interaction with the responsible regulator of each country, while considering all specifics of waste handling, potential facility access, and site-specific regulations in detail. The impact and the opportunities created come primarily from two distinct reasons: a) the significantly increased fuel utilization leading to reduction in waste generated per unit energy produced; and b) the innovative approach of reverse reprocessing which is only possible in a liquid fueled reactor. The latter creates the possibility of elementwise fission product separation through online salt clean-up which further opens up technical opportunities for segregation of material streams based on half-life, radioactivity, or heat load, as well as secondary demand of radioisotopes for medical or industrial applications.

The data presented here should be seen as a basis for the proposed deeper discussions within the entire waste management community, including the P&T and final disposal specialists as well as the regulator(s) responsible for the waste strategies in each country. Potential national-level discussions could be adapted from some of the key points raised during recent deliberations in Germany [78], including:

- Could different repositories be built or used/extended for long-lived waste separated from heat-generating waste?
- How to handle heat-generating fission products – long-term interim storage for 500 years to 1000 years or repurposing of material after an initial cooling period of 50 years instead of permanent disposal? Could this be combined with the interim storage which will anyway be needed due to the delay in the site selection process?
- How to handle negligible heat-producing, long-lived waste – what long-term behaviour can be expected, and could long-lived waste be treated like LLW or ILW with appropriate conditioning?
- What changes to licenses and permits of existing waste disposal facilities, if any, would be necessary?
- What are the differences between national waste classification and management framework to those followed internationally? Is there a need to rationalize the country-specific criteria by aligning it to international standards?

Discussions such as these will be crucial to evaluate the new potential opportunities for reimagining nuclear waste management strategies which have been created through the breakthrough technology of iMAGINE. This novel approach has opened new potential, but it can only be leveraged in a collaborative approach where the key stakeholders must come together to solve the challenge along with the required dedication and investment for development and demonstration. Many specialists may consider geological final disposal to be a shorter and more cost-effective solution for the high-level waste problem. However, higher cost efficiency does not always correspond to more sustainability, a key driving factor in the modern world. Moreover, considering that the origin of current waste management strategies dates to the time of the big landfills of the 1970s, as well as the time and investment required for a future geological disposal facility, it would be worth discussing and investigating such an innovative technological solution in parallel.

## Conclusions

Globally, there has been a growing recognition of nuclear energy as a key enabler in solving the energy trilemma and transitioning to a decarbonized society. However, societal concerns about long-term nuclear waste management have been a key impediment in gaining widespread public acceptance and support for the technology. The waste disposal problem was foreseen since the earliest days and closed fuel cycle with P&T was later proposed as the desired solution. Although many key steps of P&T have been demonstrated at laboratory scales, it has never matured to industrial levels due to excessive costs, complexity, and proliferation risks. Above all, P&T has not been able to deliver on its main promise – to avoid the demand for the long-term, high-level waste disposal.

iMAGINE, a chloride molten salt fast reactor based technology using reverse reprocessing, has been proposed to overcome the remaining challenges offering a holistic cradle-to-grave system-level solution. The opportunities created through iMAGINE for reducing the deep geological disposal challenge have been investigated in this paper and the analysis complements earlier works on long-term operational studies of iMAGINE. The long-lived fission products generated by the system and the resultant radiological hazard were evaluated. These were analyzed with respect to the waste management strategies currently followed in some of the major nuclear power producing countries. The insights presented here should be seen as the basis for deeper discussions between the waste management and P&T community, the final disposal community, and the related regulators responsible for the waste management strategies in the countries to evaluate the opportunities. It is important to note that the waste classification and management criteria vary significantly from country to country with only one parameter being decisive in some (for example, only the decay heat in Germany) while others use a combination of more than one quantity (for example, specific activity and dose rate in India, or specific activity and decay heat in France), as discussed in the Nuclear Waste Classification and Management section.

It has been shown that a uniform worldwide analysis leading to meaningful results is not possible and thus, investigations must be performed on country specific criteria. The results have shown that even with highly conservative and simplifying assumptions, only Sn^121m^, Cs^137^ and Sm^151^ with half-lives less than 100 years exceed the respective national thresholds and, thus, would require deep geological disposal in many countries. In fact, for France and Sweden, even Cs^137^ would be excluded due to its half-life being below 31 years. The countries with the most stringent classification and disposal criteria are Russia followed by India and Finland. This leads to all 10 significant long-lived fission products (with half-lives varying from 30 to ~ 10 million years) being considered as HLW requiring deep geological disposal in India and Russia. However, I^129^ does not meet the necessary thresholds for deep disposal in Finland. Interestingly, as per the stipulated criteria in Japan, disposal in deep underground facilities is only required for Tc^99^ and I^129^ which is different from most of the other countries. Finally, based on the available information, concrete conclusions cannot be drawn for the UK and USA. It must be reiterated here that these results are highly conservative and do not consider the different optimization strategies, such as using prolonged interim storage or modern conditioning methods.

Although the country-specific results and disposal requirements may vary, it can be concluded that iMAGINE drastically simplifies the long-term nuclear waste management challenge by greatly reducing the amount of HLW requiring deep geological disposal. In fact, under suitable conditions, iMAGINE will most probably be able to deliver on the promises of P&T for certain countries, potentially allowing a limited time storage system instead of the long-term high-level waste disposal. This is achieved by significantly improved fuel utilization leading to significantly reduced waste generation per unit energy produced. This is due to the design of iMAGINE as an iso-breeder with online clean-up to remove important neutron poisoning fission products instead of fissile materials, which also makes the system proliferation resistant as no fissile material must be separated. Moreover, the staggered online salt clean-up in iMAGINE enables elementwise fission product separation and related storage and conditioning. This creates novel technical opportunities for segregation of materials based on half-life, radioactivity or heat load allowing tailored conditioning of different waste streams, as well as serving the secondary demand of radioisotopes for medical or industrial applications.

The results presented here should be seen as the basis for the proposed deeper inter-disciplinary discussions between the relevant stakeholders in various countries. These discussions should evaluate the new possibilities for reimagining nuclear waste management, addressing a key impediment in the widespread acceptance and success of nuclear energy technologies through the breakthrough technology of iMAGINE. This novel approach has opened new potential, but it can only be leveraged through a closely coordinated collaborative effort for developing and demonstrating an advanced technology with sustained dedication and investment. iMAGINE offers the opportunity of a paradigm shift in long-term nuclear waste management away from the current strategies originating back to the time of big landfills of 1970s. In addition, considering the time and investment required for a future geological disposal facility, it would be worth discussing and investigating this novel innovative approach in parallel.
